# National Diabetes Month — November 2017

**DOI:** 10.15585/mmwr.mm6643a1

**Published:** 2017-11-03

November is National Diabetes Month. Approximately 114 million U.S. persons are living with diabetes (30 million) or prediabetes (84 million) (*1*). Persons with prediabetes are at increased risk for developing type 2 diabetes, heart disease, and stroke (*1*). Type 2 diabetes can be prevented through lifestyle changes (e.g., weight loss, healthy eating, and increased physical activity) (*1*,*2*). Persons with diabetes can take steps to control the disease and prevent complications (*1*,*3*). This issue of MMWR includes a report on diabetes-related kidney failure.

Working with partners, CDC plays an important role in preventing or delaying the onset of type 2 diabetes, preventing complications of diabetes, and improving health and quality of life for persons with diabetes. The National Diabetes Statistics Report, 2017 (*1*) provides the latest statistics about diabetes. With the Ad Council, the American Diabetes Association, and the American Medical Association, CDC has developed public service announcements to encourage persons to take the prediabetes risk test (https://DoIHavePrediabetes.org). CDC also joined forces with CBS Television Stations in a television and digital miniseries, “Your Health with Joan Lunden and CDC,” to provide information about diabetes prevention and control (https://www.cdc.gov/diabetestv/index.html). More information is available at https://www.cdc.gov/diabetes.

## References

[1] CDC. National diabetes statistics report, 2017. Atlanta, GA: US Department of Health and Human Services, CDC; 2017. https://www.cdc.gov/diabetes/data/statistics/statistics-report.html

[2] Knowler WC, Barrett-Connor E, Fowler SE, ; Diabetes Prevention Program Research Group. Reduction in the incidence of type 2 diabetes with lifestyle intervention or metformin. N Engl J Med 2002;346:393–403. 10.1056/NEJMoa012512 11832527 PMC1370926

[3] Venkat Narayan KM, Williams D, Gregg EW, Cowie C, eds. Diabetes public health: from data to policy. New York, NY: Oxford University Press; 2011.

